# Supplementary material for: A Unique Isolation of a Lytic Bacteriophage Infected Bacillus anthracis Isolate from Pafuri, South Africa
Source: Microorganisms. 2020 Jun 20;8(6):932. doi: 10.3390/microorganisms8060932 (PMC7356010; doi:10.3390/microorganisms8060932)
Supplement: Supplementary file 1 [file microorganisms-08-00932-s001.zip › microorganisms-804784-supplementary/Supplementary files/supplementary material.docx]

**Table 1.** To determine the *Bacillus* phage Crookii effects on *Bacillus anthracis* in decomposing blood, Romanowsky-Giemsa stained smears were visually appraised microscopically at three time points. The bacterial counts (vegetative cells versus endospores) were enumerated under different conditions (standard incubation, carbon dioxide incubation, sodium bicarbonate content and bacteriophage type) for comparison. Counts represent cells/spores per 100µL of blood.

| **Host *Bacillus anthracis* bacterium** | **Infection by Bacteriophage** | **Standard Incubation** | **8% CO_2_ Incubation** | **0.8% w/v NaHCO_3_** | **8 hours** | | **12 hours** | | **24 hours** | |
| --- | --- | --- | --- | --- | --- | --- | --- | --- | --- | --- |
|  |  |  |  |  | Vegetative cell count | Spores and Endospore count | Vegetative cell count | Spores and Endospore count | Vegetative cell count | Spores and Endospore count |
| Sterne | None | X |  |  | 950 | 0 | ∞ | 0 | *∞ | *∞ |
| DS201579 | None | X |  |  | 720 | 0 | ∞ | 0 | *∞ | *∞ |
| Sterne | Gamma | X |  |  | 551 | 14 | 118 | 193 | 0 | 137 |
| Sterne | Crookii | X |  |  | 410 | 32 | 157 | 123 | 0 | 56 |
| DS201579 | Crookii | X |  |  | 368 | 24 | 26 | 32 | 0 | 11 |
| Sterne | None |  | X |  | 786 | 0 | ∞ | 968 | *∞ | *∞ |
| DS201579 | None |  | X |  | 587 | 0 | ∞ | 852 | *∞ | *∞ |
| Sterne | Gamma |  | X |  | 523 | 182 | 197 | 215 | 0 | 168 |
| Sterne | Crookii |  | X |  | 366 | 162 | 83 | 151 | 0 | 89 |
| DS201579 | Crookii |  | X |  | 168 | 124 | 15 | 58 | 0 | 64 |
| Sterne | None | X |  | X | 80 | 36 | 125 | 321 | 0 | 513 |
| DS201579 | None | X |  | X | 46 | 21 | 112 | 202 | 0 | 296 |
| Sterne | Gamma | X |  | X | 0 | 45 | 0 | 32 | 0 | 35 |
| Sterne | Crookii | X |  | X | 0 | 50 | 0 | 41 | 0 | 34 |
| DS201579 | Crookii | X |  | X | 0 | 65 | 0 | 36 | 0 | 29 |
| Sterne | None |  | X | X | 9 | 115 | 0 | 130 | 0 | 124 |
| DS201579 | None |  | X | X | 6 | 121 | 0 | 122 | 0 | 128 |
| Sterne | Gamma |  | X | X | 5 | 78 | 0 | 79 | 0 | 168 |
| Sterne | Crookii |  | X | X | 5 | 92 | 0 | 85 | 0 | 79 |
| DS201579 | Crookii |  | X | X | 12 | 86 | 0 | 74 | 0 | 66 |

∞ = cells are too numerous to count; *∞ = too numerous to count but equally abundant vegetative cell count = intact bacterial cells without evidence of white endospore contained within.

**Table 2.** General features of the genome sequence of *Bacillus anthracis* DS201579 and *Bacillus* phage Crookii.

| **Features** | ***B. anthracis* DS201579** | | | **PHAGE_Crookii** |
| --- | --- | --- | --- | --- |
| Domain | Bacteria | | | Virus |
| Replicon | Chromosome | Plasmid pX01 | Plasmid pX02 |  |
| Sequence reads after trim | 3 670 390 | | | 1 734 240 |
| Avg. length after trim | 222 | 222 | 222 | 143 |
| Genome size (bp) | 5 176 511 | 178 546 | 94 997 | 154 012 |
| Number of contigs | 26 | 4 | 1 | 1 |
| Maximum length | 1 768 393 | 6 6440 | - | - |
| Minimum length | 1 385 | 4 4636 | - | - |
| G+C content (%) | 35 | 32 | 33 | 37 |
| Number of coding sequences | 5 724 | 198 | 127 | 235 |
| Number of RNAs | 89 | 0 | 0 | 0 |
| Number of tRNA | 75 | 0 | 0 | 2 |
| Prophages | 5 | - | - | - |

**Table 3.** Five prophages of *Bacillus anthracis* DS201579 in the chromosome identified using PHASTER.

| **Region** | **Region Length (Kb)** | **Completeness** | **Score** | **# Total Proteins** | **Region Position** | **Most Common Phage** | **GC %** | **% of Similarities*** | **Ames ancestor Prophages** |  |
| --- | --- | --- | --- | --- | --- | --- | --- | --- | --- | --- |
| LVWF01000002.1, *Bacillus anthracis* strain DS201579,DS201579_contig_1 | | | | | | | | | | |
| 1 | 65.1 | questionable | 90 | 87 | 570686-635837 | PHAGE_Bacill_1_NC_009737 | 34.67% | 12.64 | LambdaBa04 |  |
| 2 | 51.9 | incomplete | 50 | 77 | 860374-912282 | PHAGE_Bacill_PfEFR_5_NC_031055 | 35.14% | 40.25 | PHAGE_Bacill_PfEFR_5_NC_031055 |  |
| LVWF01000007.1, *Bacillus anthracis* strain DS201579, DS201579_contig_14 | | | | | | | | | | |
| 3 | 45.8 | intact | 110 | 57 | 150522-196327 | PHAGE_Bacill_phBC6A52_NC_004821 | 35.28% | 17.54 | LambdaBa01 |  |
| LVWF01000024.1, *Bacillus anthracis* strain DS201579, DS201579_contig_3 | | | | | | | | | | |
| 4 | 14.6 | questionable | 70 | 16 | 188669-203313 | PHAGE_Lactob_Ldl1_NC_026609 | 36.02% | 31.25 | LambdaBa03 |  |
| LVWF01000029.1, *Bacillus anthracis* strain DS201579,DS201579_contig_7 | | | | | | | | | | |
| 5 | 8.7 | incomplete | 30 | 13 | 144045-152762 | PHAGE_Bacill_WBeta_NC_007734 | 34.15% | 38.46 | LambdaBa02 |  |

Intact (score > 90); Questionable (score 70-90); Incomplete (score < 70); *The percentage of proteins in # phage hit proteins that are most similar to the most common phage proteins.

Table S4: *De novo* assembly of unmapped reads collected from read mapping analysis of *Bacillus anthracis* DS201579 using *B. anthracis* Ames Ancestor as a reference genome (GenBank accession: NC_007530.2; NC_007322.2 and NC_007323,2). See attachment (too large to fit and remain legible)


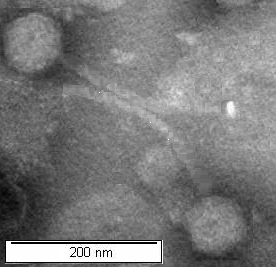


**Figure 1.** Transmission electron micrographs of bacteriophage *Bacillus* phage Crookii, isolated from an anthrax carcass site in Kruger National Park, South Africa. It was negatively stained with 2% uranyl acetate. *Bacillus* phage Crookii has a *Myoviridae* morphology with a thickened contractile tail in relation to head. The head is ~80nm with a tail measuring ~205nm.


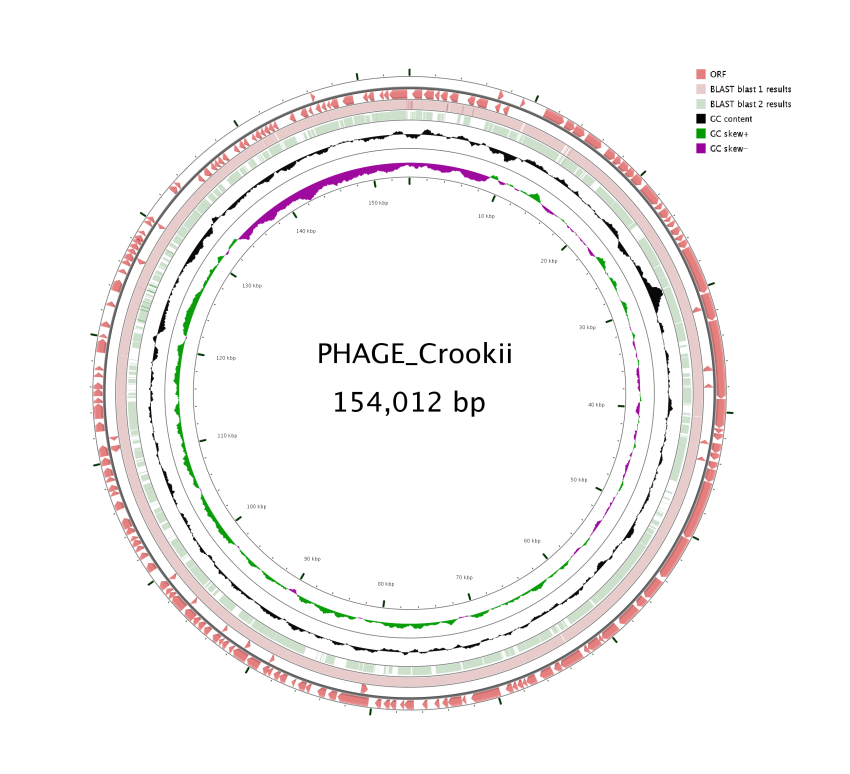


**Figure 2.** The CGView comparative tool map of *Bacillus* phage Crookii genome sequence to *Bacillus* phage WPh. Circles display from inside to outside: genomic position in kilobases, GC skew (purple and green), GC content (black). Blast 1 results show the sequence homolog between *Bacillus* phage Crookii and phage WPh (blast 2). Open reading frames are indicated as pink from the outside.
