# Supplementary material for: A Unique Isolation of a Lytic Bacteriophage Infected Bacillus anthracis Isolate from Pafuri, South Africa
Source: Microorganisms. 2020 Jun 20;8(6):932. doi: 10.3390/microorganisms8060932 (PMC7356010; doi:10.3390/microorganisms8060932)
Supplement: Supplementary file 1 [file microorganisms-08-00932-s001.zip › microorganisms-804784-supplementary/Supplementary files/Table S3.docx]

**Table S3:** Five prophages of *Bacillus anthracis* DS201579 in the chromosome identified using PHASTER.

| Region | Region Length (Kb) | Completeness | Score | # Total Proteins | Region Position | Most Common Phage | GC % | % of Similarities* | Ames ancestor Prophages |
| --- | --- | --- | --- | --- | --- | --- | --- | --- | --- |
| LVWF01000002.1, *Bacillus anthracis* strain DS201579,DS201579_contig_1 | | | | | | | | | |
| 1 | 65.1 | questionable | 90 | 87 | 570686-635837 | PHAGE_Bacill_1_NC_009737 | 34.67% | 12.64 | LambdaBa04 |
| 2 | 51.9 | incomplete | 50 | 77 | 860374-912282 | PHAGE_Bacill_PfEFR_5_NC_031055 | 35.14% | 40.25 | PHAGE_Bacill_PfEFR_5_NC_031055 |
| LVWF01000007.1, *Bacillus anthracis* strain DS201579, DS201579_contig_14 | | | | | | | | | |
| 3 | 45.8 | intact | 110 | 57 | 150522-196327 | PHAGE_Bacill_phBC6A52_NC_004821 | 35.28% | 17.54 | LambdaBa01 |
| LVWF01000024.1, *Bacillus anthracis* strain DS201579, DS201579_contig_3 | | | | | | | | | |
| 4 | 14.6 | questionable | 70 | 16 | 188669-203313 | PHAGE_Lactob_Ldl1_NC_026609 | 36.02% | 31.25 | LambdaBa03 |
| LVWF01000029.1, *Bacillus anthracis* strain DS201579,DS201579_contig_7 | | | | | | | | | |
| 5 | 8.7 | incomplete | 30 | 13 | 144045-152762 | PHAGE_Bacill_WBeta_NC_007734 | 34.15% | 38.46 | LambdaBa02 |

Intact (score > 90)

Questionable (score 70-90)

Incomplete (score < 70)

*The percentage of proteins in # phage Hit proteins that are most similar to the most common phage proteins.
