# Supplementary figures and images for: A Unique Isolation of a Lytic Bacteriophage Infected Bacillus anthracis Isolate from Pafuri, South Africa
Source: Microorganisms. 2020 Jun 20;8(6):932. doi: 10.3390/microorganisms8060932 (PMC7356010; doi:10.3390/microorganisms8060932)

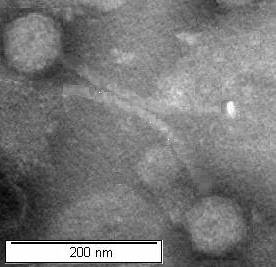

Supplement: Supplementary file 1 [file microorganisms-08-00932-s001.zip › microorganisms-804784-supplementary/Supplementary files/Figure S1.png]
